# A New Perspective on Lumbar Disc Herniation Management Using Prone Knee Extension

**DOI:** 10.1155/carm/2579261

**Published:** 2026-01-07

**Authors:** Majid Shahbazi, Tayyebeh Sadat Fatemi

**Affiliations:** ^1^ Department of Physiotherapy, School of Paramedical and Rehabilitation Sciences, Mashhad University of Medical Sciences, Mashhad, Iran, mums.ac.ir; ^2^ Ward of Physiotherapy, Imam Reza Hospital, Mashhad University of Medical Sciences, Mashhad, Iran, mums.ac.ir

**Keywords:** lumbar disc herniation, pain management, physical therapy, prone knee extension

## Abstract

**Introduction:**

Lumbar disc herniation (LDH) is a frequent cause of low back pain and radiculopathy, often resulting in diminished functional capacity and a lower quality of life. Nonsurgical interventions are frequently sought to manage symptoms and enhance spinal stability. This case report explores a novel application of the prone knee extension (PKE) exercise as part of a comprehensive physiotherapy regimen aimed at addressing pain, mobility limitations, and functional impairments in a patient with LDH.

**Case Presentation:**

A 34‐year‐old male with a history of LDH and radiating lower limb symptoms presented with an acute exacerbation following heavy physical activity. MRI confirmed a disc protrusion at the L4‐L5 level. The patient reported severe pain, restricted lumbar extension, and functional limitations. A 5‐week treatment plan was implemented, consisting of 10 physiotherapy sessions combining the PKE exercise, infrared heat therapy, dry needling, diaphragmatic breathing, and core stability exercises, alongside a home exercise program. Significant improvements were observed, including pain reduction, resolution of radiating symptoms, increased lumbar range of motion, and improved sleep quality. Functional assessments using the Oswestry Disability Index (ODI) and Global Rating of Change (GRC) Scale demonstrated reduced disability and enhanced overall function.

**Conclusion:**

This case highlights the potential effectiveness of integrating the PKE exercise into the management of LDH. The approach facilitated pain relief, improved health status, and enhanced functional capacity, suggesting that it could serve as a valuable nonsurgical intervention in clinical practice. However, as the findings are preliminary, further research is needed to validate these results.

## 1. Introduction

It is estimated that around 80% of individuals will suffer from lower back pain at some point in their lives [1, 2]. Lumbar disc herniations (LDHs) are a leading cause of lower back pain, affecting approximately 1%–3% of the population each year, with the condition most commonly seen in individuals between the ages of 30 and 50 years [3]. Compared to other areas of the spine, disc herniations are most frequently found in the lumbar region, particularly at the L4‐L5 and L5‐S1 levels [1]. LDH with radiculopathy (LDHR) occurs when the displaced disc material compresses or makes contact with the lumbar nerve roots [1]. LDHR treatment includes both nonsurgical and surgical options, with increasing efforts over the past decade to minimize spinal surgeries [1]. Surgery is generally recommended for severe cases, symptoms lasting longer than 6 weeks, failure of conservative treatment, or in the presence of neurological symptoms such as saddle anesthesia, incontinence, sudden paresis, or cauda equina syndrome [1, 4]. Studies show that about 85% of patients prefer nonsurgical management due to its lower risks and costs [5]. Conservative methods, including patient education, the McKenzie method, mobilization, manipulation, exercise therapy, and traction, are commonly recommended [1]. Exercise therapy, in particular, plays a crucial role in improving the function of lower back muscles [6].

Exercise has a broad positive effect on the clinical outcomes of LDH. It can help reduce pain [7], improve lumbar spine mobility [8], and significantly enhance range of motion (ROM) in forward flexion and backward extension. Additionally, exercise contributes to improved quality of life, mental health, and sleep [7], Encouraging patients to maintain a positive outlook while preventing any aggravation of the disease or its symptoms. Certain exercises also improve muscle coordination, flexibility, and balance, enhancing lumbar spine stability [9]. Overall, exercise interventions support rehabilitation and improve health outcomes in LDH patients [6]. Various exercises have been recommended for patients with LDH, including lumbar spine stabilization, McKenzie exercises, clinical Pilates exercises, and general exercises [7, 10, 11].

Based on our review of the current literature, no study to date has specifically investigated the effect of the prone knee extension (PKE) exercise in patients with LDH. Nevertheless, a preliminary biomechanical rationale supports its potential therapeutic value. The “flat back” deformity, characterized by reduced lumbar lordosis, is known to alter spinal alignment and increase intradiscal pressure, particularly at the L4‐L5 and L5‐S1 levels, thereby predisposing individuals to disc degeneration and symptom exacerbation [12, 13]. Within this conceptual framework, the establishment of optimal spinal alignment—characterized by the restoration of a neutral or physiological lordotic curvature—serves to mitigate intervertebral disc loading and enhance lumbopelvic stability. This biomechanical improvement subsequently correlates with potential symptomatic relief and functional recovery in patients with LDH [14]. The quadriceps muscle, particularly the rectus femoris component, plays a critical role in regulating pelvic tilt and influencing lumbar curvature. A tight rectus femoris can induce an anterior pelvic tilt, which secondarily modulates lumbar lordosis as a postural adaptation [15–17]. Furthermore, the quadriceps contributes significantly to overall spinal and lower limb stability [18]. Therefore, the PKE exercise may help restore spinal alignment, reduce disc stress, and enhance function. This case report aims to explore the therapeutic potential of the PKE exercise for a patient with LDH, addressing a notable gap in the existing clinical literature.

## 2. Case Study

### 2.1. Case Presentation

#### 2.1.1. History

The 34‐year‐old male patient, weighing 84 kg and standing 178 cm tall, works as service personnel in an office and has no history of underlying medical conditions. The patient reported no previous participation in organized sports or regular athletic training programs. He experienced two episodes of acute low back pain (LBP) in the year prior to starting this intervention. The first acute episode, approximately 1 year earlier, began after lifting heavy objects and resulted in LBP and right leg pain localized to the posterior thigh, gluteal region, heel, and occasionally numbness in the toes. To manage these symptoms, a neurologist prescribed Gabapentin 300 mg and Naproxen 250 mg for pain and inflammation, along with therapeutic exercises such as one‐sided trunk movements, oblique sit‐ups, and prone hip extensions. These interventions reduced the pain, though mild recurrences occasionally occurred due to work activities.

The second acute episode occurred approximately 6 months ago when the pain intensified in the lower lumbar region after the patient lifted his 20 kg child from the ground in the morning, while fully bending forward at the trunk. The patient reported acute pain with subsequent difficulty in movement and rested for 1 month after the lifting incident. During this phase, he experienced severe pain, especially in the left lower limb, with sensations resembling knife‐like stabbing pain. The pain was so severe that he was unable to sleep for several nights and also experienced lateral thigh numbness. The day before the second acute episode, the patient had driven continuously for about 5 h, and at that time, he also experienced some pain in the lower back. A magnetic resonance imaging (MRI) was performed (Figure 1), revealing disc protrusions at the L4‐L5 and L3‐L4 levels with central bulging. Medications prescribed included Mixodin, Nuvazin, and Profen, which provided some relief. Following the acute phase, morning stiffness and coldness in the left lateral calf persisted, but after walking for about 5 min, the pain decreased. The patient also began experiencing increased discomfort in the left big toe, which worsened with prolonged sitting and driving. Upon sitting and driving, the patient experienced pain in the right gluteal region, which radiated down to the left ankle. Fear of running was also noted.

Figure 1Sagittal (a) and transverse (b) views of the spine, demonstrating disc protrusions at the L4‐L5 and L3‐L4 levels with central bulging. These images illustrate the extent of the disc protrusions and their relationship to surrounding spinal structures.

**Figure:**
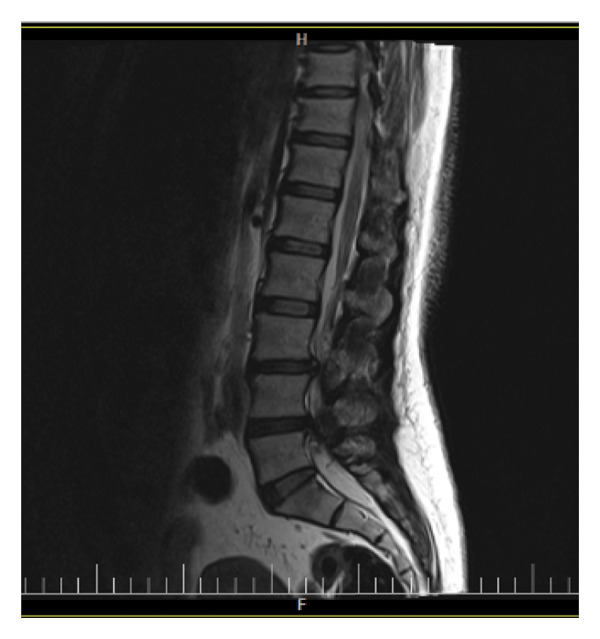
(a)

**Figure:**
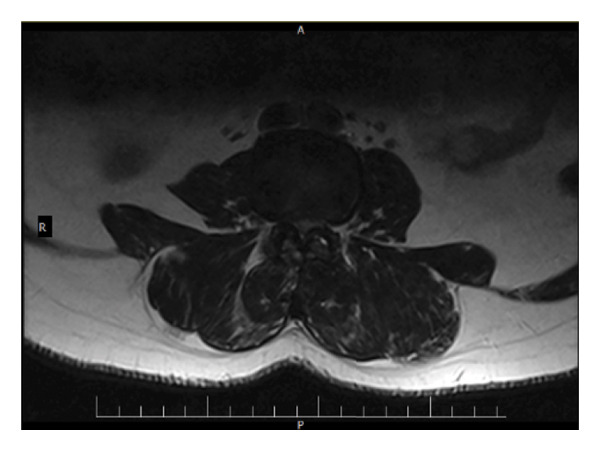
(b)

#### 2.1.2. Clinical Examination

Tenderness graded as 3/4 was observed in the 5th and 4th lumbar regions [19]. Palpation of the quadratus lumborum revealed tenderness graded as 2/4, eliciting symptoms, particularly on the left side. While performing the straight leg raise (SLR) test at an angle of approximately 75°, the patient reported pain rated as 4/10 on the Numerical Pain Rating Scale (NPRS) in the lower back and along the path of the left sciatic nerve [19]. Standing lumbar extension was painful at 15  of ROM, with pain reported as 6/10 on the NPRS. During standing lumbar flexion, the patient rated the pain as 3/10 on the NPRS, and ROM was full [20]. All other lumbar movements were painless with full ROM. The sacroiliac joint tests were negative [19].

#### 2.1.3. Outcomes Measurement

The primary outcomes for this case report included the patient’s self‐reported pain, measured using the NPRS, a tool that has demonstrated good reliability and responsiveness in populations with LBP [21]. Functional disability was assessed with the Oswestry Disability Index (ODI) [22], which has reported strong internal consistency (Cronbach’s *α* = 0.828) and high test–retest reliability (ICC = 0.871) [23]. The patient’s perception of improvement was evaluated using the Global Rating of Change (GRC) Scale, which measures overall changes in the patient’s condition over time [24] and has shown excellent test–retest reliability in individuals with LBP [25]. Lumbar spine ROM was also assessed, focusing on flexion and extension movements, using a goniometer [20], a method that has demonstrated good reliability and validity in clinical settings [26]. Data were collected at four stages: baseline (prior to the intervention), after the 5th session, after the 10th session, and 3 months posttreatment. For ROM measurements, the patient was instructed to perform each motion to their maximum capability without inducing additional pain.

#### 2.1.4. Treatment

**Figure 3:**
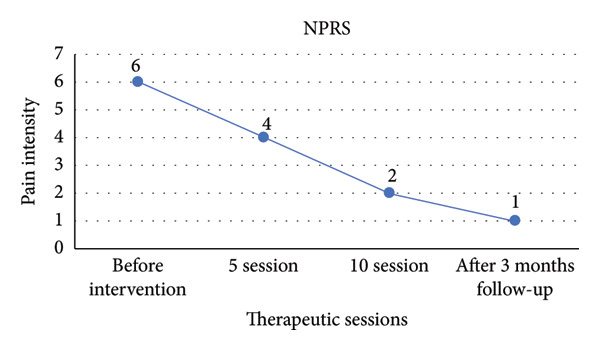
Progression of pain reduction across four sessions.

**Figure 4:**
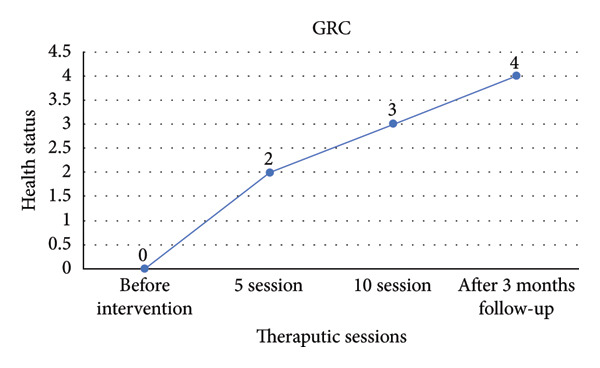
Patient’s perceived improvement over time as assessed by the Global Rating of Change (GRC) Scale.

The patient underwent a physiotherapy program consisting of 10 sessions over 5 weeks, with two sessions per week. Each session lasted 40 min and included the PKE exercise for 15–20 min combined with diaphragmatic breathing, tailored to the patient’s tolerance and fatigue level. Additionally, each session incorporated dry needling and 20 min of superficial infrared heat therapy [27]. Ethical principles were strictly adhered to in this interventional case report. Informed consent was obtained after providing comprehensive details about the study’s objectives, intervention type, risks, benefits, and data usage. Confidentiality was ensured, and no identifying information was disclosed without explicit consent. Patient safety was closely monitored, and the intervention was immediately discontinued if any serious risks arose. The intervention was based on scientific evidence and designed to minimize unnecessary risks. Previous studies have demonstrated that lumbar extension exercises can improve spinal alignment, reduce pain, and enhance function in patients with LDH [28, 29]. Participation was voluntary, and the patient retained the right to withdraw at any time without affecting access to standard treatment. During the first two sessions, pain levels and the patient’s ROM were assessed immediately after the exercises to evaluate the independent effect of PKE, separate from dry needling, and to ensure no adverse effects from the exercise. The patient was advised to refrain from using analgesics unless symptoms worsened and was educated on avoiding activities that could exacerbate symptoms, particularly in the early treatment phases. For home exercise, PKE exercises were prescribed twice daily, with each session lasting 10 min. Following the completion of the 10 sessions, the patient underwent a general lumbar manipulation due to localized pain (rated 2/10 on the NPRS) at the L5‐S1 junction, which subsequently improved. By the end of the 10th session, following a significant reduction in the patient’s pain, core stability exercises were introduced alongside the PKE exercises. The patient continued to perform this combination of exercises until the fourth evaluation stage [30]. The PKE exercise progression was systematically tailored for patients requiring reduced pressure on anterior spinal elements, such as those with LDH. The stages included (1) bilateral PKE with a neutral lumbar spine (Figures 2(a), 2(b)); (2) bilateral PKE with mild lumbar extension, achieved by placing a single pillow approximately 10 cm thick under the chest (Figures 2(c), 2(d)); (3) bilateral PKE with moderate lumbar extension, achieved by placing two pillows approximately 20 cm thick under the chest (Figures 2(e), 2(f)); (4) bilateral PKE with moderate lumbar extension and upper limbs in flexion, targeting latissimus dorsi stretching to enhance lumbar lordosis (Figures 2(g), 2(h)); and (5) bilateral PKE with posterior knee weight (5 kg), moderate lumbar extension, and upper limbs in flexion, designed to further activate the quadriceps and hip adductors, thereby promoting lumbar lordosis and stability (Figures 2(i), 2(j)). Progression occurred every other session based on the absence of symptom exacerbation, which was monitored through subjective pain reports, functional assessments, and observation of radiating symptoms.

Figure 2Exercise progression used in the study: (a) and (b) show bilateral prone knee extension with lumbar neutral position, (c) and (d) depict bilateral prone knee extension with mild lumbar extension, (e) and (f) demonstrate bilateral prone knee extension with moderate lumbar extension, (g) and (h) illustrate bilateral prone knee extension with moderate lumbar extension and upper limbs in flexion position, and (i) and (j) represent bilateral prone knee extension with posterior knee weight (5 kg), moderate lumbar extension, and upper limbs in flexion position.

**Figure:**
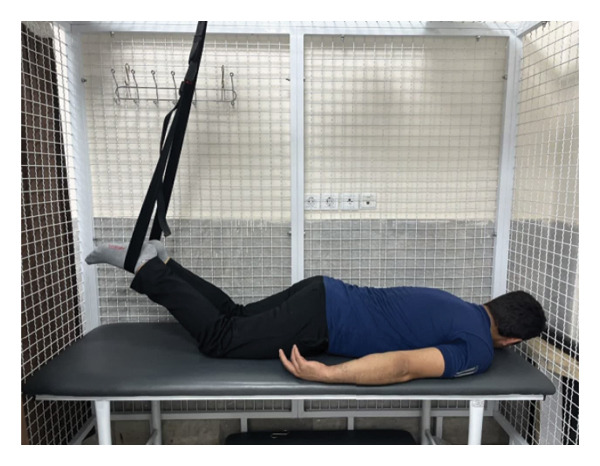
(a)

**Figure:**
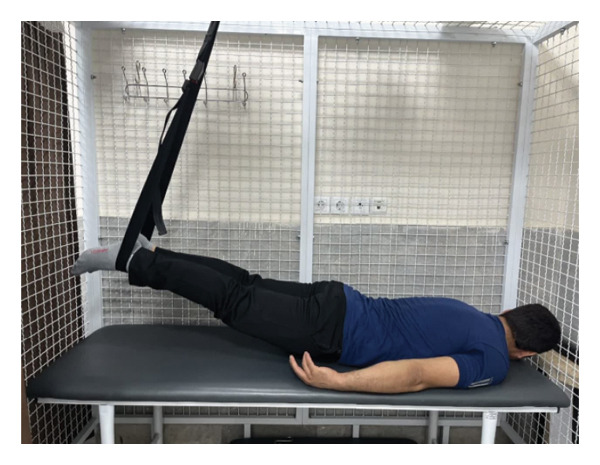
(b)

**Figure:**
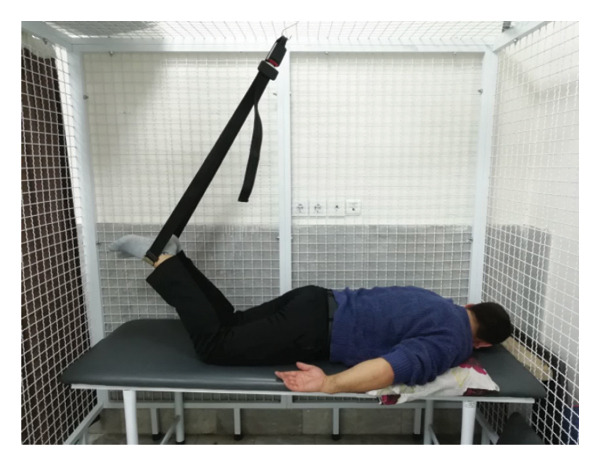
(c)

**Figure:**
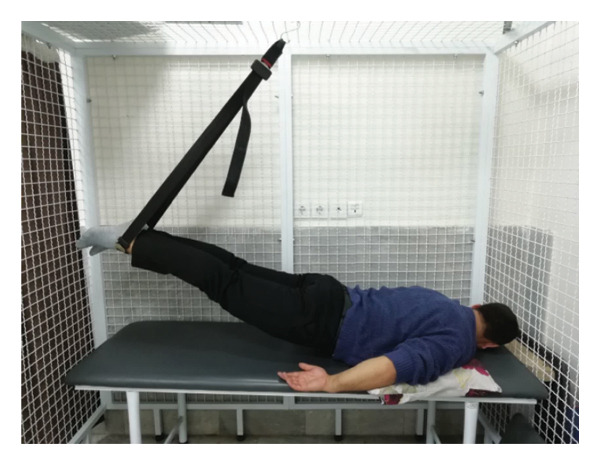
(d)

**Figure:**
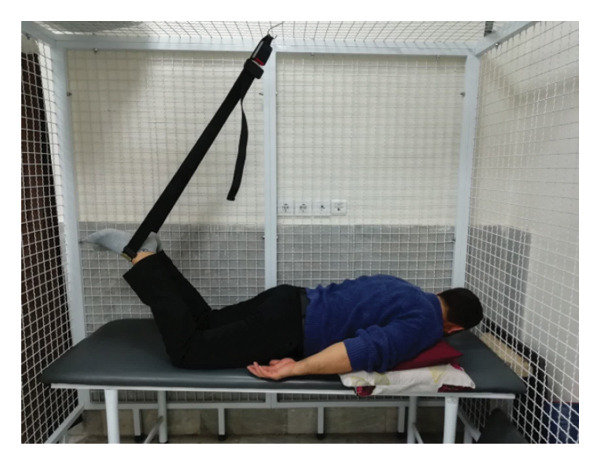
(e)

**Figure:**
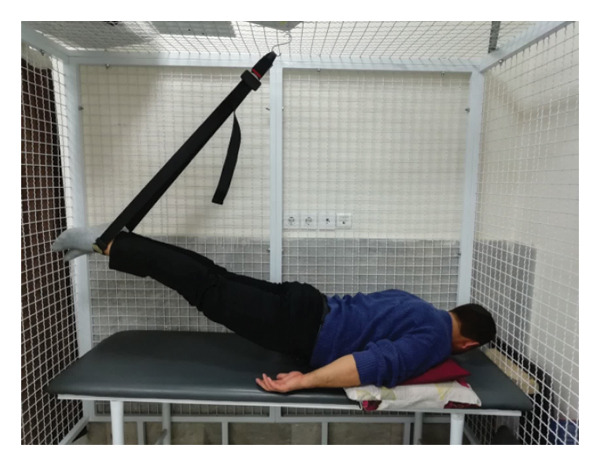
(f)

**Figure:**
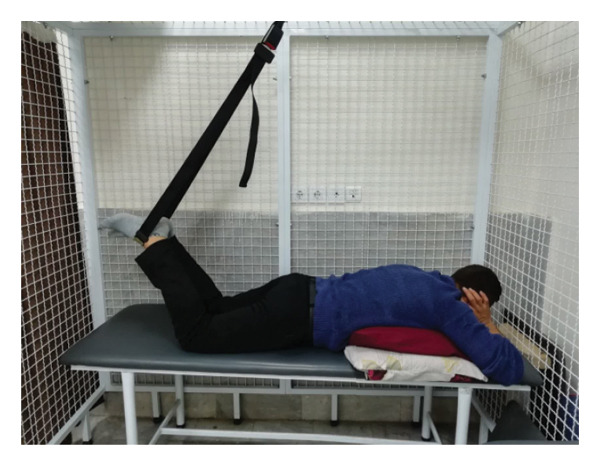
(g)

**Figure:**
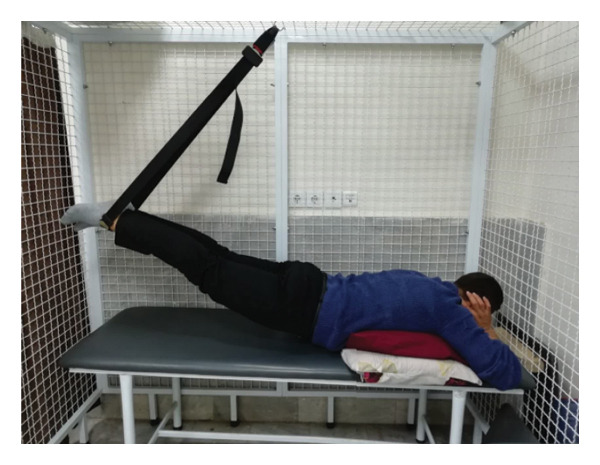
(h)

**Figure:**
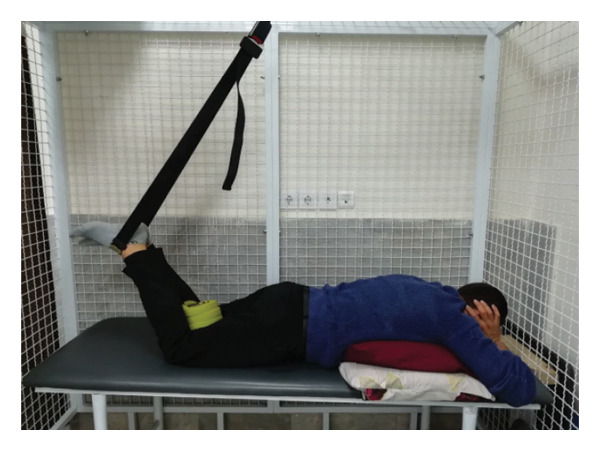
(i)

**Figure:**
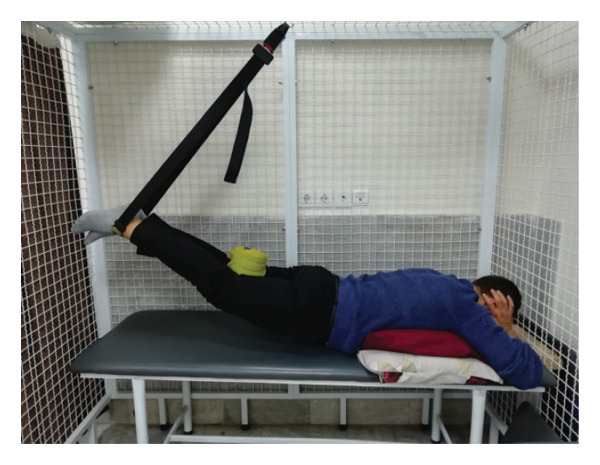
(j)

## 3. Results

The study’s results demonstrated significant improvements in the patient following the interventions. By the end of the 5th session, the patient’s lumbar flexion ROM was fully restored and pain‐free. The lumbar extension ROM, initially limited to 15°, improved to 25° without pain. Additionally, the SLR test returned to normal. ROM was objectively measured using a manual goniometer. Tenderness on palpation of the L4 and L5 vertebrae and the quadratus lumborum showed significant improvement, as qualitatively assessed by the clinician. The patient also reported substantial reductions in sitting‐related discomfort and associated pain in the right hip, leg, and heel. Burning and tingling sensations in the right leg were markedly reduced, alongside a noticeable improvement in sleep quality. Pain in the left ankle, most prominently on the dorsum, significantly decreased. Sensory disturbances and cold sensations in the left foot also improved over the course of treatment. These outcomes were corroborated by pain reduction data collected across four sessions and visualized in the accompanying chart (Figure 3).

The GRC Scale assessed the patient’s perceived improvement over time, showing significant progress (Figure 4).

The patient showed significant improvement across all domains of the ODI, with a marked reduction in both pain and functional limitations, as shown in Table 1. The progression of disability over the 4‐month period indicates that the implemented therapeutic interventions played a key role in the patient’s recovery, demonstrating the effectiveness of the treatment approach in managing chronic pain and enhancing functional outcomes.

**Table 1:** Details of ODI in a patient with low back disc herniation.

ODI item	Before intervention	5th session	10th session	After 4‐month follow‐up
Pain intensity	3	2	1	1
Personal care (washing, dressing, etc.)	1	0	0	0
Lifting	4	3	2	1
Walking	2	1	1	0
Sitting	4	2	1	1
Standing	5	3	1	1
Sleeping	3	2	1	0
Sex life (if applicable)	1	1	0	0
Social life	2	2	0	0
Traveling	3	2	1	1
Total	28	18	8	5
ODI (%)	56	36	16	10

## 4. Discussion

The case report highlights the effectiveness of therapeutic exercise, specifically PKE, in improving outcomes for a patient with LDH. Pain‐free flexion was fully restored, lumbar extension improved significantly, and sensory disturbances, pain, and related symptoms were notably reduced. Improvements in the GRC and ODI scores further demonstrate the success of the treatment in enhancing mobility, reducing pain, and improving overall functionality. It is important to clarify that stabilization training is recommended as a subsequent phase after restoring mobility in patients with LDH to ensure long‐term spinal support and functional recovery. This approach is supported by previous studies demonstrating that core stabilization exercises can improve spinal alignment and motor control and reduce recurrence of LBP [31, 32].

The findings of this case report align with previous studies that have utilized lumbar extension exercises to promote lumbar extension and increase lumbar lordosis in the management of LDH [33–35]. However, some studies suggest that lumbar extension exercises may not always improve symptoms in LDH patients and could, in some cases, exacerbate pain or provide limited functional benefits [35–37]. Several factors may explain the observed outcomes. First, during PKE, the pelvis and lumbar spine are positioned to support the natural lumbar lordosis [38]. Compressive forces on the intervertebral discs are greater in lumbar spines with reduced lordosis compared to those with increased lordosis. However, forces on the facet joints are higher in spines with excessive lordosis [39]. By increasing lumbar lordosis and relieving pressure on the intervertebral discs, PKE helps to reduce discomfort from disc herniation [40]. Additionally, strengthening the quadriceps is another key factor. These muscles play a vital role in stabilizing the pelvis and lower spine. By enhancing quadriceps strength through exercises like PKE, the muscles provide better support to the lumbar spine, reducing pressure on the discs and alleviating pain [41]. The rectus femoris muscle, which contributes to anterior pelvic tilt, can further increase lumbar lordosis [42]. Reduced lumbar lordosis, characteristic of a “flat back” posture, is associated with increased risk of disc degeneration, particularly at the L4‐L5 and L5‐S1 levels [43]. Exercises targeting the quadriceps and improving lumbar lordosis may thus help alleviate LDH symptoms by enhancing spinal alignment, reducing strain on the discs, and providing long‐term relief. As quadriceps strength improves, pelvic and lumbar spine stability also improves, contributing to better spinal function and pain reduction. It seems that, unlike Mackenzie’s exercise, which involves correction from cranial to caudal, this corrective exercise is performed from caudal to cranial. In addition to these biomechanical effects, the progression of PKE exercises may also provide neurophysiological benefits through the principles of graded exposure and movement‐directed therapy. Emerging evidence increasingly supports movement‐directed therapy and graded exposure as key components of effective management, prioritizing functional restoration and reduction of pain‐related fear over static alignment alone [44]. Since lumbar extension initially produced mild discomfort, the gradual increase in movement intensity likely reduced fear of movement, improved neuromuscular control, and promoted pain desensitization, thereby enhancing functional recovery [45, 46]. Furthermore, movement‐directed therapy restores adaptive motor patterns and functional re‐engagement by encouraging controlled, nociceptive‐tolerant motion. This framework diminishes fear‐avoidance behaviors and cognitions, alleviates movement‐related anxieties, and cultivates adaptive coping strategies alongside self‐efficacy [47]. Consequently, PKE likely normalized spinal kinematics, optimized lumbopelvic coordination, and fostered confident, asymptomatic mobility—pivotal elements of enduring recovery in LDH.

## 5. Limitation

This case report has several limitations. First, the findings cannot be generalized to a wider population, as they are based on a single patient’s specific condition and may differ in other individuals. Second, the absence of a control group limits the ability to determine the true effects of the intervention. Moreover, the concurrent use of additional therapeutic interventions makes it difficult to attribute the observed improvements solely to the PKE exercise. Additionally, reliance on the patient’s self‐reported outcomes may have introduced bias influenced by psychological or cognitive factors. Finally, the same clinician conducted both the treatment and outcome assessments, which could have introduced assessor bias. However, this potential bias was minimized through the use of standardized, objective measurement tools and consistent evaluation procedures. These limitations highlight the need for larger, well‐controlled studies to confirm and isolate the specific effects of the intervention.

## 6. Conclusion

The findings of this study suggest that incorporating the PKE exercise into a comprehensive rehabilitation program may be beneficial for patients with lumbar discopathy, as evidenced by associated improvements in pain and functional outcomes. While the specific contribution of PKE cannot be isolated with certainty due to the presence of cointerventions, the observed gains are consistent with the proposed biomechanical rationale for this exercise, which may contribute to improved lumbopelvic stability, strengthening the quadriceps muscles and enhancing lumbar lordosis. Further research with a more controlled design is warranted to isolate the definitive effect of PKE. More research with a more exact design and a bigger sample size is required to generalize these findings.

## Ethics Statement

As this study is a case report based on a single patient, formal ethical approval was not required according to the guidelines of Mashhad University of Medical Sciences.

## Consent

Informed consent was obtained from the patient for participation in this case report and for the publication of clinical information.

## Conflicts of Interest

The authors declare no conflicts of interest.

## Funding

No funding was received for the completion of this case report.

## Data Availability

The data that support the findings of this study are available on request from the corresponding author. The data are not publicly available due to privacy or ethical restrictions.
